# MACE in the Race: A Canadian Perspective on Major Adverse Cardiac Events (MACE) During Running

**DOI:** 10.7759/cureus.28323

**Published:** 2022-08-23

**Authors:** Mohammed Abrahim

**Affiliations:** 1 Division of Emergency Medicine, Department of Family Medicine, McMaster University, Hamilton, CAN

**Keywords:** long-distance running, premature cardiac death, exercise-induced myocardial infarction, marathon, sudden cardiac death (scd), major adverse cardiac effect (mace)

## Abstract

The recent tragic deaths of two fit and healthy Canadian physicians during running have shocked the whole Canadian medical community. In order to prevent such loss of precious human lives, the paradox of dying during a life-prolonging activity begets further contemplation and investigation on whether we have been missing something in assessing the risk of major cardiovascular adverse events (MACE) in fit individuals during long-distance running. Additionally, knowing the potential, yet the rare fatal risk of running, physicians are obliged to disclose that fatal risk while prescribing exercise to their patients according to the Supreme Court of Canada Ruling. Further research is urgently needed.

## Editorial

In July 2022, within one week, two physically fit Canadian physicians died suddenly after sustaining a major adverse cardiac event (MACE) while long-distance running (LDR) [1,2]. Paradoxically, the activity that is supposedly intended to prolong an individual’s life became the major factor responsible for their death. Such paradox begets further contemplation and investigation on whether we have been missing something in assessing the risk of MACE in fit individuals during LDR to avoid further loss of precious human lives.

Historically, the root of long-distance or marathon running dates back to 490 BC, during the Persian invasion of Greece [3]. The legend has it that Phidippides, a 40-year-old Athenian man, ran 26.2 miles from the town of Marathon to Athens to deliver the victory news. However, once he reached Athens he collapsed and died. Instead of declaring long-distance running as the cause of Phidippides’ demise, the distance he ran was established as the official marathon race distance [3]. Phidippides became the earliest recorded case of a fatal MACE during LDR.

In recent decades, LDR has become a mainstream sport, rather than once considered extreme and exclusively the domain of a small group of ultra-athletes. This change was partly instigated through the work of Jim Fixx, a prominent figure in the fitness scene back in the 1970s [3]. Fixx wrote the best-selling book, The Complete Book of Running. Sadly, he suffered a fatal MACE, secondary to coronary atherosclerosis, during LDR at the age of 52 [3].

MACEs are defined as myocardial infarction (MI), stroke, heart failure, and/or death from other cardiovascular diseases [4]. The most common cause of exercise-induced MACE is MI with atherosclerosis being the most common aetiopathogenesis of MIs [5]. Alarmingly, to date, the only credible and gold standard diagnostic modality of atherosclerosis is arterial catheterization which is both invasive and costly and impedes its wide application among individuals and/or the healthcare system [5]. In the absence of an objective method of diagnosing atherosclerosis, autopsy studies have provided more accurate insight into the prevalence of this disease, particularly among young individuals [6]. Atherosclerosis has been labeled as a disease of the old, inactive, and obese [5]. However, in a postmortem investigation of the coronary blood vessels in the bodies of those killed in the Vietnam war, who were lean and physically active soldiers with an average age of 22 years old, 50% showed evidence of atherosclerosis [6].

One of the earliest autopsy studies investigating fatal MACE during LDR was performed in California between 1973 and 1978 and identified 18 cases, the study was limited to the deaths when the autopsy was performed with the vast majority with MI as their recorded cause of death [7]. In a larger study of fatal MACE among athletes in the United States between 1980 and 2006, 1,866 cases were identified, and again MACE was the most common cause of death [8].

At present, it is unclear what the rates of MACE induced by LDR in the general population are, because research has been focused on a small population of athletes who registered and participated in official LDR events. But MACE in regular individuals who decide to adopt a “healthy” lifestyle by LDR fail to be included in the statistics of LDR MACE.

Undoubtedly, there is a plethora of scientific evidence supporting the beneficial impact of physical exercise on longevity [9]. The current physical activity guidelines for adult Americans recommend at least 150 minutes to 300 minutes a week of moderate-intensity, 75 minutes to 150 minutes a week of vigorous-intensity aerobic physical activity, or an equivalent combination of moderate- and vigorous-intensity aerobic activity [9]. Physicians are encouraged to prescribe exercise as primary prevention not only to fit individuals but also for those cardiometabolic disorders [10]. However, if physicians are ought to prescribe exercise, like all other treatments, we are required to master not only the therapeutic dose of the proposed treatment but also its safety margin including the adverse effects and its toxic or fatal dose. Moreover, the Supreme Court of Canada affirmed that even if a treatment risk is “a mere possibility,” yet it carries with it serious consequences such as death or paralysis, it should be disclosed [11]. Therefore, when prescribing exercise, we ought to disclose its rare but potentially fatal and non-fatal MACE risk, precisely because there is no guarantee that physically fit and active individuals are protected against MACE during LDR.

To conclude, there is a sizeable and growing body of evidence on the incidence of fatal and non-fatal MACE during LDR among professional athletes during official LDR. On the other hand, there is a paucity of research investigating the incidence of MACE during habitual vigorous exercise, such as LDR. Until the development of an objective atherosclerosis test, physicians should be careful when prescribing exercise without disclosing that it carries with it an unlikely potential to induce fatal and non-fatal MACE. With the increasing popularity of LDR, there is an urgent need to assess the safety of such sport and clearly define the at-risk population. To increase the surveillance of such a potentially fatal and disabling condition, physicians filling in the cause of death in death certificates should make a clear distinction between the immediate, antecedent, and underlying cause of death. Finally, it is essential that physicians inform their patients not only about the health benefits of physical exercise but also about the potentially life-threatening risk of that activity among certain populations. By doing so, we may be able to reduce the mortality and disability associated with MACE.
